# Are Anticapsular Antibodies the Primary Mechanism of Protection against Invasive Pneumococcal Disease?

**DOI:** 10.1371/journal.pmed.0020015

**Published:** 2005-01-25

**Authors:** Marc Lipsitch, Cynthia G Whitney, Elizabeth Zell, Tarja Kaijalainen, Ron Dagan, Richard Malley

**Affiliations:** **1**Departments of Epidemiology and Immunology and Infectious Diseases, Harvard School of Public HealthBoston, MassachusettsUnited States of America; **2**Active Bacterial Core Surveillance and Division of Bacterial and Mycotic Diseases, National Center for Infectious DiseasesCenters for Disease Control and Prevention, Atlanta, GeorgiaUnited States of America; **3**National Reference Laboratory for Pneumococcus, National Public Health InstituteOuluFinland; **4**Pediatric Infectious Disease Unit, Soroka University Medical Center and the Faculty of Health Sciences, Ben-Gurion University of the NegevBeer-ShevaIsrael; **5**Children's Hospital and Harvard Medical SchoolBoston, MassachusettsUnited States of America; University of LondonUnited Kingdom

## Abstract

**Background:**

Antibody to capsular polysaccharide has been the basis of several vaccines that offer protection against invasive disease from Streptococcus pneumoniae. The success of such vaccines has led to the inference that natural protection against invasive pneumococcal disease is largely conferred by anticapsular antibody. If this is so, one would expect that the decline in disease from different serotypes would vary significantly, and that the appearance of substantial concentrations of anticapsular antibodies would coincide temporally with the decline in age-specific incidence.

**Methods and Findings:**

Using incidence data from the United States, we show that, on the contrary, the decline in incidence with age is quite similar for the seven most important serogroups, despite large differences in exposure in the population. Moreover, only modest increases in antibody concentration occur over the second and third years of life, a period in which serotype-specific incidence declines to less than 25% of its peak. We also present detailed data on the distribution of antibody concentrations in Israeli toddlers, which are consistent with the United States findings. The same conclusion is supported by new data on age-specific incidence in Finland, which is compared with published data on antibody acquisition in Finnish toddlers.

**Conclusion:**

We suggest some additional studies of the mechanisms of protection that could distinguish among potential alternative mechanisms, including acquired immunity to noncapsular antigens, maturation of nonspecific immune responses, or changes in anatomy or exposure.

## Abstract 2

Anticapsular antibodies induced by vaccination can protect against invasive streptococcal disease, but natural immunity seems to involve other mechanism as well

## Introduction

The protective effects of antibody to pneumococcal capsular polysaccharides have been appreciated since the development of serum therapy, in which passively transferred, serotype-specific antipneumococcal serum reduced mortality from pneumococcal pneumonia by half [1]. The development of pneumococcal polysaccharide vaccines for adults [2] and the efficacy of pneumococcal polysaccharide–protein conjugate vaccines in infants and children [3,4] have confirmed that active immunity to the polysaccharide can provide excellent protection against invasive disease from pneumococci of the same serotype, and in some cases protection against cross-reacting serotypes within the same serogroup.

While the ability of passive or vaccine-induced anticapsular antibodies to protect against pneumococcal disease is clear, less is known about the natural development of immunity to pneumococcal disease in unimmunized persons. In unimmunized populations, the incidence of invasive disease follows a well-known age distribution, peaking in the first 2 y of life, declining by more than an order of magnitude by the second and third decades of life, and then rising at an accelerating pace, with incidence in persons over 70 y approaching that in infants [5]. The reason for the decline in incidence has not been conclusively determined, yet it is often suggested that the acquisition of anticapsular antibodies plays a critical role in this decline [6,7]. Indeed, it has been proposed that the human immune system sees each serotype of Streptococcus pneumoniae as a distinct, independent pathogen [8].

The hypothesis that protection from invasive pneumococcal disease is caused by the acquisition of anticapsular antibodies directed against each of the pneumococcal serotypes yields two simple predictions about the age-specific epidemiology of pneumococcal disease. First, it predicts that the age-specific timing of the decline in invasive disease should be different for different serotypes: those that are rare, poorly immunogenic, or both should decline later in life than those that are common and immunogenic. Second, it predicts that protection against invasive disease from a given serotype should coincide temporally with the acquisition of anticapsular antibody to that serotype, both at an individual level and at a population level. We tested these predictions using data from the United States, Finland, and Israel.

## Methods

### United States Dataset

Incidence of invasive pneumococcal disease was measured in eight sites around the United States participating in the Centers for Disease Control and Prevention's Active Bacterial Core Surveillance between 1994 and 1999. The data used here are restricted to those periods during which serotyping was routinely performed: 1994–1999 for the Georgia site, 1995–1999 for the Minnesota site, and 1998–1999 for all other sites [5]. Data were not available on the timing of anticapsular antibody acquisition in these same populations, but we compared the timing of the decline in pneumococcal disease against previously published data on age-specific prevalence of anticapsular antibody levels greater than 0.2 mcg/ml [9].

### Israel Dataset

Antibody concentrations were measured by enzyme-linked immunosorbent assay (ELISA) (with absorption by cell wall polysaccharide but not by 22F polysaccharide) in blood samples that were obtained from 130 toddlers at enrollment and at approximately 12 and 24 mo after enrollment in a double-blind, controlled trial of a nine-valent pneumococcal conjugate vaccine. The toddlers analyzed for this study were those in the control group, which received meningococcal C conjugate vaccine; the details of the trial [10] were previously described. Preliminary analyses of these data confirmed previous findings [11] that ELISA measurements were highly correlated (and therefore likely revealed cross-reactions) for all pairs of serotypes, except for type 14, for which correlations were minimal, consistent with previous findings of little cross-reaction. For this reason, we chose to analyze age trends only in serotype 14 antibodies.

### Finland Dataset

Mandatory reporting from all microbiological laboratories in Finland to the National Register of Infectious Disease (http://www3.ktl.fi/stat/) identified all blood and cerebrospinal fluid isolates of S. pneumoniae obtained in the years 1995–2001. Incidence within 6-mo age groups was calculated using population denominators obtained from Statistics Finland (Helsinki, Finland). Since the primary purpose of examining incidence in Finland was to compare age-specific rates against published distributions of antibody concentrations for the same age groups [12], we restricted our attention to serotype 14 and serogroup 6, for which subsequent investigations suggested antibody measurements in Finland were relatively unaffected by cross-reactions [13] (ELISA measurements for published data from Finland used a special type 6B polysaccharide that was found to minimize cross-reactions [13]).

## Results

### United States Findings

**Figure 1:**
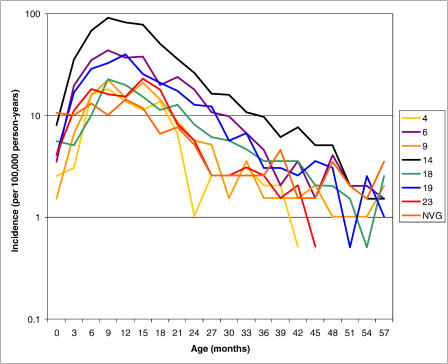
Age-Specific Incidence of Invasive Pneumococcal Disease in the United States by Serogroup, Based on Data from Active Bacterial Core Surveillance Serogroups 4 and 23 are shown only up to 48 mo, after which incidence is less than 1/100,000 person-years. All serogroups besides those in the heptavalent vaccine are shown combined as non-vaccine serogroups (NVG).

**Figure 2:**
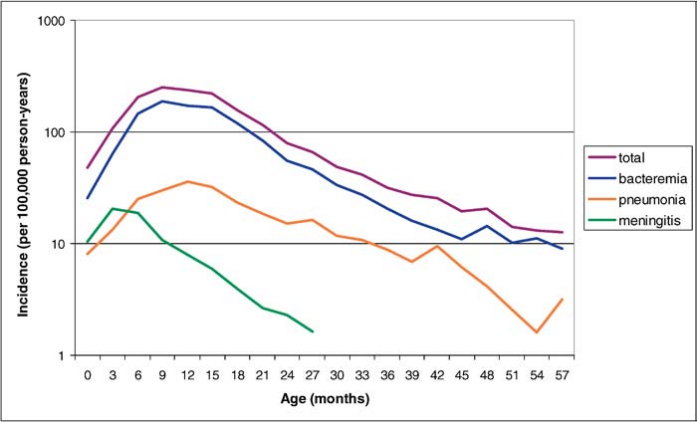
Age-Specific Incidence of Invasive Pneumococcal Disease in the United States by Disease Type, Based on Data from Active Bacterial Core Surveillance Meningitis incidence is plotted only up to 30 mo, after which it remains at or below 1/100,000 person-years. “Pneumonia” indicates bacteremic pneumonia, while “bacteremia” indicates nonfocal bacteremia. “Total” includes other invasive diagnoses.

Figure 1 shows the age-specific incidence of invasive pneumococcal disease, by capsular serogroup, obtained from population-based active surveillance in the United States prior to the introduction of the conjugate vaccine. Figure 2 shows age-specific incidence by type of infection, for the same age range.

Incidence peaks between the ages of 9 and 15 mo, and falls in an approximately parallel fashion thereafter, for each of the seven most important serogroups (which are those included in the seven-valent conjugate vaccine) and for the remaining serogroups put together. The same pattern is observed for both pneumonia and bacteremia. For each serogroup, incidence by age 24 mo is approximately half that in the peak age group, and by 36 mo, incidence for each serogroup has fallen to 10%–25% of its peak.

The consistent timing of the pattern across multiple serogroups argues for a common mechanism, rather than for independent acquisition of immunity to each serogroup as a separate event. Since most individuals do not suffer from invasive pneumococcal disease in this age range, carriage or mucosal disease (otitis media) from pneumococci may be the immunizing event for anticapsular antibodies in the general population [12] (although in principle immunity to some serogroups could be generated in response to cross-reacting antigens from other bacterial species or other sources [14]). Different serogroups have vastly different frequencies among pneumococci isolated from carriage [12,15,16,17] and otitis media [12,18]; for example, serogroups 4 and 18 and the non-vaccine serogroups are isolated far less commonly than several of the other pneumococcal types identified in Figure 1. One could postulate that these differences in frequency of carriage are offset by differences in immunogenicity; however, there is little evidence that serotypes 4 or 18C are more immunogenic than other, far more common serotypes [4,15]. One could also postulate that the frequency of isolation of serotypes from carriage depends on duration as well as incidence, so that the serotypes for which carriage appears rare are simply carried for a shorter duration. While the data to address this speculation are limited, the duration of carriage of types 4 and 18C seems to be comparable to that of other, more frequently carried serotypes [15,16]. Thus, the most parsimonious interpretation of the data on the timing of the decline in age-specific susceptibility is that one or more common mechanisms are responsible for the decline in disease from all serotypes.

Testing the second prediction against data is hampered by the fact that, to our knowledge, no study has characterized the age-specific distribution of antibody concentration in a large population using the currently accepted methodology, which includes absorption with both cell wall polysaccharide and serotype 22F polysaccharide [13,19]. Analyses by Soininen and colleagues have found that antibodies measured by standard ELISA in unimmunized children are highly cross-reactive between different serotypes, and that cross-reactive antibodies lack opsonophagocytic function and often appear in the absence of any documented exposure to a given capsular serotype. As a result, age-specific antibody concentration data for any given serotype are “contaminated,” to a greater or lesser degree, by cross-reactive antibodies with other specificties.

The most important exception to this problem occurs for antibodies to serotype 14, for which cross-reaction is minimal [11]. A recent publication describes the age-specific proportion of children in the United States with anti-type-14 polysaccharide antibody concentration exceeding the putative protective concentration of 0.2 μg/ml (Figure 3 of [9]). At 12 mo, 90%–95% of the population falls below this level, and at 24 mo, 80%–85% remains below it—despite a 40%–50% drop in disease incidence from 12 mo to 24 mo. At 36 mo, 75% of children remain below the putative protective level, although by this age incidence has fallen more than 80% from its 12-mo peak. In summary, if the 0.2-μg/ml concentration were truly the threshold for “protection,” the 20%–30% reduction in the unprotected population between ages 12 and 36 mo would be inadequate to explain the 90% decline in disease incidence. Clearly, 0.2 μg/ml is not a precise dividing line between being “protected” and “unprotected,” a threshold that (if it exists) may vary by serotype, but given the available data, there is reason to doubt that anti-type-14 antibody alone is responsible for the decline in disease in this age range.

**Figure 3:**
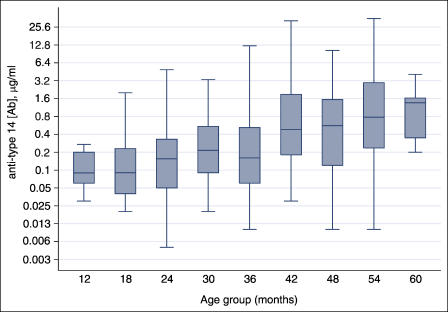
Box-Whisker Plot of Anti-Type-14 Polysaccharide Antibodies in Israeli Toddlers, by 6-Mo Age Groups Central boxes indicate median and 25th and 75th percentiles; whiskers indicate upper and lower adjacent values.

### Israel Findings

To assess whether the limitations of the United States antibody described above—i.e., the availability of only one cutoff point for antibody concentrations—might be providing an incomplete picture of the distribution of antibody levels by age, we examined an additional dataset from Israeli toddlers. For the reasons described above, we examined only antibodies to serotype 14, for which the distribution of concentrations by age is shown in Figure 3. These data indicate that between the ages of 12–17 and 36–41 mo, the median antibody concentration increases by about 2-fold. These data are broadly consistent with those published for the United States; antibody levels rise very gradually, though detectably, during the second and third years of life. It is difficult to believe—albeit not impossible—that the dramatic declines in disease incidence over these years are explained simply by this small rise in antibody concentrations.

### Finland Findings

**Figure 4:**
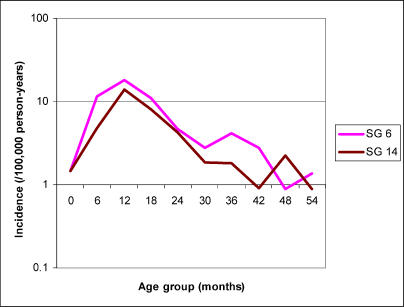
Age-Specific Incidence of Invasive Pneumococcal Disease Caused by Serogroups 6 and 14 in Finland, Based on Active Laboratory-Based Surveillance

Incidence of serogroup 6 and serotype 14 invasive pneumococcal disease by 6-mo age groups in Finland, shown in Figure 4, is broadly similar to that found in the United States, albeit with lower absolute incidence for both serogroups. Peak incidence occurs in the 12–17-mo age group, and incidence declines to 25%–30% of its peak rate by 24–29 mo of age. This decline in incidence may be compared against the cumulative distributions of antibody concentrations in Finnish toddlers shown in Figure 2 of [12]. Between ages 12 and 24 mo, there is a discernible increase in the concentration of antibodies in the population, but the median concentration increases by only about 2-fold in this period. Moreover, the proportion of the population with antibody concentration below any particular threshold that may indicate protection changes little in this period. For example, the proportion of the population with anti-type-14 antibody concentrations less than 0.2 μg/ml declines from approximately 55% to approximately 40%, and the proportion with less than 0.5 μg/ml is reduced from about 95% to about 80%. Similar patterns are seen in the Finnish antibody data for type 6B [12]. Thus, as in the Israeli data, only a very small shift in the distribution of type-specific anti-polysaccharide antibody concentration is observed during the second year of life, yet incidence of invasive disease from the serotypes in question declines substantially.

## Discussion

We have assessed two lines of epidemiological evidence, analyzed ecologically, that bear on the role of anticapsular polysaccharide antibody as the determinant of protection against invasive disease that develops during the second and third year of life. The simultaneous and approximately parallel nature of the decline in disease incidence for the seven most important serogroups in the United States suggests that one mechanism, rather than seven independent mechanisms, account for the declines in invasive disease from these serogroups. Moreover, only a slight increase in anticapsular antibody concentration is measurable in Finnish, United States, and Israeli toddlers during the same age range. As we discuss below, each of these lines of evidence is subject to caveats, but we believe that, taken together, these observations make a strong case for the importance of one or more factors other than acquisition of anticapsular antibodies in the development of protection against pneumococcal disease.

There are several possible candidates for mechanisms that could explain this age-related decline in pneumococcal disease. These include the following: acquisition of antibodies or cellular immune responses to noncapsular pneumococcal “species” antigens; age-related changes in host biology that are not related to acquired immunity, such as maturation of the innate immune system or changes in anatomy or receptors for pneumococcal attachment; changes in other risk factors, such as exposure; or changes related to other microorganisms, including changes in the resident flora or changes in the incidence of viral infections.

Systemic antibodies to several pneumococcal protein antigens, which are conserved across pneumococcal strains and serotypes, develop following pneumococcal carriage and otitis media and are present by the beginning of the second year of life [20,21]. In both Finland [20] and Kenya [21], there is an increase in the concentration of antibodies to the pneumococcal proteins pneumolysin and pneumococcal surface protein A over the first two or more years of life. In Kenya, antibodies to another conserved protein, pneumococcal surface adhesin A, showed similar distributions in the first, second, and subsequent years of life, while in Finland, levels of these antibodies were already high (equivalent to adult levels) in the first year of life, and increased above these levels in the second year. In mice, either passively transferred human serum IgG against pneumococcal surface protein A or vaccine-induced antibodies to pneumococcal surface protein A and/or pneumolysin are protective against invasive disease. Such data are consistent with the hypothesis that antibodies to these, or perhaps other, conserved pneumococcal proteins are in part responsible for the decline in invasive disease in the second and subsequent years of life.

A number of investigators have tested the hypothesis that antibodies to the pneumococcal teichoic acid, known as cell wall polysaccharide (CWPS), are capable of protecting individuals against pneumococcal invasive disease. While studies in animals [22] and humans [23] have failed to find a protective effect of antibodies to CWPS or its components, a recent study showed that passive transfer of human IgG against phosphorylcholine, a component of CWPS, could protect mice against invasive pneumococcal infections [24]. Notably, such antibodies might be elicited by a number of bacteria in addition to pneumococci, such as *Haemophilus influenzae,* which also produce phosphorylcholine. We are unaware of studies on the timing of acquisition of anti-CWPS antibodies.

We have recently shown that mice that are exposed thrice at weekly intervals to intranasal colonization with encapsulated pneumococci are protected against subsequent carriage, that this protection is effective for heterologous as well as homologous capsular types, and that it is effective even in MuMT mice, which lack the ability to produce antibodies (Malley R, Trzcinski K, Srivastava A, Thompson CM, Anderson PW, et al., unpublished data). We have also shown that intranasal immunization with unencapsulated, killed pneumococci protects against nasopharyngeal colonization, in a fashion that is independent of antibody but requires CD4+ T cells at the time of challenge. The relevance of cellular immune mechanisms in protecting humans against pneumococcal colonization or disease is not known.

Another candidate for a factor that may be changing with age is susceptibility to viral infections, especially influenza, which may predispose to pneumococcal colonization [25] or disease [26,27]. Recent evidence from clinical trials of pneumococcal conjugate vaccines shows that the vaccines can reduce the incidence of infections such as bronchiolitis that are usually associated with viruses [28] and of documented, virus-associated pneumonia [27]. These findings raise the possibility that the decline in pneumococcal disease with age reflects, in part, a decline in the incidence or severity of viral infections, so that fewer such infections lead to secondary pneumococcal disease.

Exposure to pneumococci probably changes in some fashion over the first 5 y of life. However, for changes in exposure to account for the sharp drop in disease incidence following the first birthday, it would be necessary for exposure also to drop severalfold per year over this age range. Studies of pneumococcal carriage do show gradual changes in the prevalence and serotype composition of the nasopharyngeal flora in these years, but the prevalence of carriage changes much more gradually than the incidence of invasive disease [29].

We are not aware of data that bear strongly on the plausibility of other possible mechanisms for the age-related decline in pneumococcal disease, such as changes in anatomy, physiology, receptor expression, or resident bacterial flora. However, factors other than antibody—such as innate or acquired cellular immune responses, age-related anatomical changes, or changes in exposure to pneumococci—cannot be ruled out, and more than one factor may be involved. Indeed, the peak of pneumococcal meningitis incidence in the 3–6-mo age group (see Figure 2) suggests that the mechanism of protection against meningitis may differ from those against pneumonia and bacteremia.

Although we suggest that anticapsular antibody is not primarily responsible for the age-specific decline in invasive pneumococcal disease, there is no question that the capsule is an important virulence factor that interacts with the innate and acquired immune system in a number of ways. It is clear that the pneumococcal capsule interferes with various host clearance mechanisms [30]. It would be unsurprising if different capsular types were differentially effective in permitting pneumococci to evade phagocytosis and other host defenses [31] (M. Melin, H. Jarva, S. Meri, and H. Käyhty, unpublished data). If this were the case, then one could envision that certain capsular types might in fact follow a different age-specific incidence. In particular, recent analyses suggest that serotypes 1 and 5 have relatively stable incidence over a range of age groups (W. P. Hausdorff, D. R. Feikin, and K. P. Klugman, unpublished data).

The evidence adduced here is subject to several limitations. With respect to the relative timing of acquisition of protection against different serotypes, one could postulate that because some of the most common pneumococcal serotypes, such as 6B, 19F, and 23F, are also among the least immunogenic [12], the effective exposure of the immune system is more consistent across serogroups than it appears from serogroup frequency alone. However, this pattern is not general; for example, serotype 14 is both very common and highly immunogenic [12]. With respect to the absolute timing of protection relative to the acquisition of antibody, one could argue that low levels of anticapsular antibody, perhaps of low affinity, may be present and even active at levels below those that can be reliably detected by current assays, or that B cell memory may be present and protective at an earlier age than that at which high levels of antibody are measurable. Inferences about protective antibody concentrations from animal studies and from concentrations achieved by vaccines suffer from several uncertainties. Making allowances for all of these limitations, we nonetheless believe the data suggest that mechanisms other than anticapsular antibody are primarily responsible for the age-specific decline in pneumococcal invasive disease that starts at the age of 1 y.

The likelihood that mechanisms other than anticapsular antibody confer immunity to pneumococcal disease has important implications with respect to vaccine design. As experience with conjugate pneumococcal vaccines in children unfolds, it is becoming increasingly clear that such a strategy suffers from several limitations, including the possibility of serotype replacement (already confirmed in several clinical trials), a modest effect on nasopharyngeal colonization, limited serotype coverage, cost, and difficulties in production that have led to shortages since licensure. A better understanding of the mechanisms that underlie natural immunity to pneumococcus could pave the way for the development of more effective, species-specific pneumococcal vaccines.

**Patient Summary**

**Background**

Streptococcus pneumoniae is a common bacterium that lives in the upper respiratory tract of many children, and some adults. The bacterium generally causes no harm in healthy individuals, but in some circumstances it can cause mild infections, such as ear infections, or more severe ones, such as lung infection (pneumonia), bloodstream infection (bacteremia), or infection of the lining of the brain (meningitis). These more severe forms, called invasive pneumococcal disease, occur especially in children, elderly people, and others with weakened immune systems. The bacterium exists in different versions, or serotypes. The different versions of the bacterium each have a different outer shell (the so-called bacterial capsule). Scientists have developed vaccines against Streptococcus pneumoniae that protect against the most common serotypes. These vaccines consist of a cocktail made up of material from the capsules of the most common serotypes. This material causes the body's immune system to produce antibodies that can fight Streptococcus pnemoniae and protect vaccinated individuals against disease caused by the common serotypes. In many developed countries vaccination is recommended for all children and elderly people.

**Why Was This Study Done?**

Most people get exposed to many different versions of the bacterium over the course of their lives. These encounters cause little or no disease in most people, and the risk of disease declines sharply and remains low through middle age, before climbing again in the elderly. Based on experience with vaccines, scientists have thought that this “natural” protection that develops with age was also based on antibodies against the bacterial capsule. The authors of this study wanted to test whether this was actually true.

**What Did the Researchers Do?**

If in the healthy population protection against invasive disease is in fact due to anticapsular antibodies, one can make certain predictions about the frequency of invasive disease among certain age groups. The researchers tested those predictions against actual disease records from the United States, Israel, and Finland.

**What Did They Find?**

The actual records did not match the predictions very well, suggesting that natural protection against invasive pneumococcal disease is not based on anticapsular antibodies alone.

**What Does This Mean?**

These results suggest that there are elements of natural protection against invasive pneumococcal disease that we do not understand yet. Moreover, these elements seem to involve more general protection against various forms of the bacterium rather than individual protection against particular serotypes.

**What Next?**

Given the importance of the disease, we should try to understand all elements of natural protection. Such understanding might help researchers develop better vaccines to prevent invasive pneumococcal disease, and maybe even improve treatment of patients who have become ill.

**More Information Online**

World Health Organization information page on pneumococcal vaccines: http://www.who.int/vaccines/en/pneumococcus.shtml

United States Centers for Disease Control and Prevention factsheet on pneumococcal vaccine: http://www.cdc.gov/nip/publications/VIS/vis-PneumoConjugate.pdf

Health Canada information on pneumococcal vaccine: http://www.hc-sc.gc.ca/english/iyh/medical/pneumococcal.html

Information for health-care providers from the United Kingdom Nation-al Health Service: http://www.prodigy.nhs.uk/guidance.asp?gt=Immunizations%20-%20pneumococcal

PneumoADIP Web page on childhood pneumococcal disease: http://www.pneumoadip.com/

## Abbreviations

CWPS: cell wall polysaccharide
ELISA: enzyme-linked immunosorbent assay
